# Metabolic cost of physical rehabilitation in mechanically ventilated patients in critical care: an observational study

**DOI:** 10.1136/bmjresp-2024-002878

**Published:** 2025-04-05

**Authors:** Timothy O Jenkins, Dan Stieper Karbing, Stephen Edward Rees, Mathias Krogh Poulsen, Brijesh V Patel, Michael I Polkey, Vicky MacBean

**Affiliations:** 1 Royal Brompton and Harefield Hospitals, London, UK; 2 Department of Health Science and Technology, Aalborg Universitet, Aalborg, Denmark; 3 Intramedic, Aalborg, Denmark; 4 Division of Anaesthetics, Pain Medicine and Intensive Care, Imperial College London Division of Surgery Oncology Reproductive Biology and Anaesthetics, London, UK; 5 Department of Respiratory Medicine, Royal Brompton Hospital and National Heart and Lung Institute, London, UK; 6 College of Health, Medicine and Life Sciences, Brunel University of London, London, UK

**Keywords:** Exercise, Critical Care

## Abstract

**Background:**

Physical rehabilitation is advocated to improve muscle strength and function after critical illness, yet interventional studies have reported inconsistent benefits. A greater insight into patients’ physiological response to exercise may provide an option to prescribe individualised, targeted rehabilitation, yet there is limited data measuring oxygen consumption (VO_2_) during physical rehabilitation. We aimed to test the feasibility of measuring VO_2_ during seated and standing exercise using the Beacon Caresystem and quantify within- and between-patient variability of VO_2_ percentage change.

**Methods:**

We conducted a prospective observational study on patients mechanically ventilated for ≥72 hours and able to participate in physical rehabilitation in critical care. Oxygen consumption was measured continuously using indirect calorimetry. A total of 29 measurements were taken from ten participants performing active sitting and standing exercise.

**Results:**

Median (IQR) first session baseline VO_2_ was 3.54 (2.9–3.9) mL/kg/min, increasing significantly to 4.37 (3.96–5.14) mL/kg/min during exercise (p=0.005). The median (IQR) coefficient of variation of VO_2_ percentage change in participants (n=7) who completed more than one rehabilitation session (range 2–7 sessions) was 43 (34–61)% in 26 measurements. The median (IQR) coefficient of variation of VO_2_ percentage change was 46 (26–63)% in participants performing >1 sitting exercise session (six participants, 19 sessions).

**Conclusions:**

VO_2_ increases significantly with exercise but is highly variable between participants, and in the same participant on separate occasions, performing the same functional activity. These data suggest that simplified measures of function do not necessarily relate to oxygen consumption.

**Trial registration number:**

NCT05101850.

**WHAT IS ALREADY KNOWN ON THIS TOPIC**

- Physical rehabilitation can increase oxygen consumption in mechanically ventilated patients, yet there is a paucity of evidence exploring between- and within-patient variability of oxygen consumption change in patients undertaking the same functional activity.

**WHAT THIS STUDY ADDS**

- This study shows that oxygen consumption during physical rehabilitation varies considerably within, and between, mechanically ventilated patients performing the same functional activity.

**HOW THIS STUDY MIGHT AFFECT RESEARCH, PRACTICE OR POLICY**

- These data highlight that how functional measures, such as the ICU mobility scale, do not necessarily relate to exercise intensity. Formal cardiopulmonary testing and measurement of oxygen consumption may have the potential to assist with exercise prescription and categorise rehabilitation interventions in future interventional studies.

## Introduction

 Mechanical ventilation (MV) is a life-saving intervention; however, patients who undergo MV commonly develop intensive care unit (ICU) acquired weakness.^1^ Skeletal muscle wasting occurs rapidly in mechanically ventilated patients, with data showing a 17.7% reduction in rectus femoris cross-sectional area in ten days.^2^ ICU acquired weakness is associated with increased length of hospital stay and impaired recovery,^3 4^ and patients experience loss in muscle strength and function for up to five years after critical illness.^5^

Physical rehabilitation in critical care is advocated to improve muscle strength and function.^6^ However, while feasible, most interventional studies of early or intensive rehabilitation on critical care have failed to show a consistent improvement in outcomes.^7^,^10^ Recent work even suggests that high-intensity early rehabilitation for as long as possible is associated with increased adverse events compared with normal care, with no difference in the number of days alive and out of hospital at day 180.^11^ These findings suggest that rehabilitation may not be as effective as first thought, perhaps due to over- or undertraining patients, either of which may hamper physical recovery, delay liberation from MV or cause adverse events.^11 12^

Rehabilitation ‘dose’ in clinical practice and interventional trials is often quantified as the highest functional activity achieved by the patient during the session. The ICU mobility scale^13^ is commonly used as an objective tool to quantify a patient’s function; this is a ten-point scale ranging from 0 (lying in bed with no active movement) to 10 (walking independently without a gait aid). The scale is easy to use but does not capture all aspects of physical function, including engagement, exertion and intensity.

Indirect calorimetry offers an objective measure of exercise intensity during rehabilitation in critical care as measured by oxygen consumption (VO_2_), which could aid prescription of exercise in mechanically ventilated patients.^14^ Some data exist on the metabolic response to exercise in critically ill, mechanically ventilated patients during bed exercises and bed-based cycling,^1215^,^17^ or mixed physiotherapy interventions, including passive exercise, cycling and airway clearance.^18^ Black *et al* found that, in mechanically ventilated patients, VO_2_ increased by a mean (SD) of 23.3 (11.2)% when sitting on the edge of the bed and by 34.8 (13.3)% during standing activities.^19^ Collings and Cusack do not report percentage changes but show wide 95% CIs in VO_2_ data during sitting on the edge of the bed in ten critically unwell individuals.^14^

The aims of this observational study were as follows. (1) Test the feasibility of taking indirect calorimetry measurement of VO_2_ during physical rehabilitation using the Beacon Caresystem (expressed as prevalence of technical issues in the measurement and analysis of VO_2_). (2) Measure oxygen consumption during physical rehabilitation in mechanically ventilated patients. We employed similar methodology to previous work,^14 19^ quantifying VO_2_ during sitting and standing exercise, realising the importance of replication^20^ to confirm the existing data in other studies. Furthermore, we aimed to take repeated measurements in the same participant to quantify variability in the VO_2_ response to sitting and standing exercise by calculating the coefficient of variation.

## Methods

The study was a single centre observational study performed across two cardiothoracic ICUs at the Royal Brompton and Harefield Hospitals within Guy’s and St Thomas’ NHS Foundation Trust, London, UK between October 2021 and May 2022.

### Participants

Participants were included if they were aged 18 years or over, had been invasively ventilated for ≥72 hours with an endotracheal tube or tracheostomy in situ, respiratory rate of ≤35 breaths per minute, fraction of inspired oxygen (FiO_2_) ≤0.50, cooperative and able to participate in physical rehabilitation, and if they or their representative gave informed consent/surrogate approval. Exclusion criteria were an undrained pneumothorax/pneumomediastinum, extracorporeal membrane oxygenation (ECMO), the absence of an arterial catheter for blood sampling, pregnancy, being considered unlikely to survive, or intensivist discretion that the patient was not otherwise appropriate.

### Physical rehabilitation

Participants received usual care physical rehabilitation as prescribed by the treating physiotherapist based on the patient’s medical condition, strength and physical function. Patients were encouraged to achieve their maximum level of activity in each rehabilitation session; the resulting activity was categorised into sitting exercise (sitting over the edge of the bed: ICU mobility score 3) or standing exercise (standing or transferring to a chair: ICU mobility score 4 or 5).^13^ Reasons for ceasing exercise were recorded. Rehabilitation sessions were terminated early if any of the following occurred: chest pain suggestive of ischaemia, ischaemic ECG changes, complex ectopy, second- or third-degree heart block, fall in systolic pressure>40 mmHg from resting, hypertension (>200 mmHg systolic and/or >120 mmHg diastolic) or severe desaturation (SpO_2_<85%). Participants were studied up to four days per week until they were successfully extubated, decannulated from their tracheostomy, weaned from assisted MV, repatriated to a non-participating site or ceased to obey commands. Table 1 details reasons for discontinuation of study data collection.

**Table 1:** Reasons for discontinuation of study data collection

	n (%)
Weaned from MV	8 (80)
Ceased to obey commands	2 (20)

MVmechanical ventilation

### Measurement of VO_2_

The Beacon Caresystem (Mermaid care A/S, Norresundby, Denmark) is a bedside decision support system using mathematical models powered by an individual patient’s physiology to advise on appropriate ventilator settings. The Beacon Caresystem’s breath-by-breath indirect calorimetry function was used to measure VO_2_ in this study. The device can reliably measure VO_2_ at 0.21–0.85 FiO_2_ and has shown agreement when measuring VO_2_ at 21% and 50% inspired oxygen with the E-sCOVX (GE Healthcare, Helsinki, Finland)^21^ and the QUARK RMR (COSMED, Rome, Italy)^22^ indirect calorimetry devices. The Beacon Caresystem was connected to the participant by inserting a standard side stream respiratory gas flow sensor (SPIRIT flow sensor, Adult, Artema Technology, Germany) into the ventilator circuit, close to the patient’s airway 20 min before initiation of physical rehabilitation.

**Figure 1:**
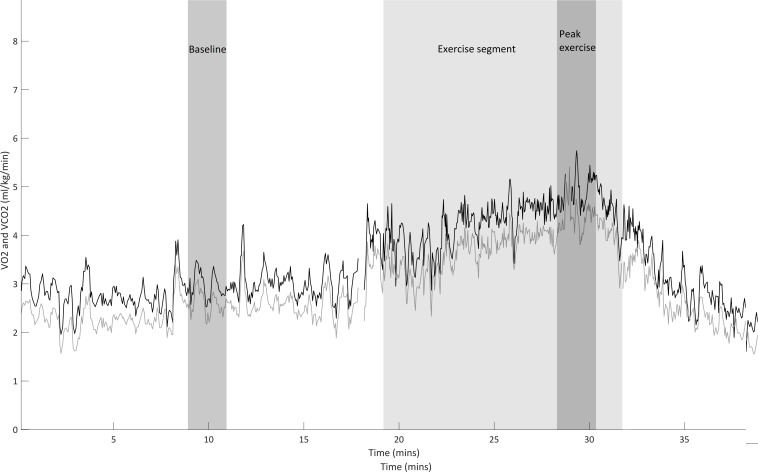
A typical participant’s VO_2_/VCO_2_ data. Black line shows the VO_2_ signal and grey line shows the VCO_2_ signal. Shaded medium grey area shows the 2-min average taken at baseline, shaded light grey area shows the exercise segment and shaded dark grey area shows the 2-min average corresponding to peak exercise.

Figure 1 shows VO_2_ and carbon dioxide production (VCO_2_) signal output from the Beacon caresystem during a typical rehabilitation session. Baseline VO_2_ (mL/kg/min) was measured as a 2 min average during a steady state of rest prior to the rehabilitation session. This was the longest steady state that could be recorded while avoiding influences such as coughing and position changes. Mean exercise VO_2_ (mL/kg/min) was calculated by dividing the total rehabilitation session VO_2_ (mL/kg) by the total activity time in minutes (from when the participant started to move to when the participant returned to supine). Percentage increase in VO_2_ attributable to exercise was calculated using mean exercise VO_2_ and baseline VO_2_. Peak VO_2_ was calculated by determining the highest 2-min VO_2_ average during the rehabilitation session.

### Sample size

No a priori sample size calculation was undertaken due to the feasibility nature of the study.

### Statistical analysis

Variables are presented as median and IQR due to the sample size. Comparison of paired data (first session baseline, change in VO_2_ and peak VO_2_) during rehabilitation was analysed using the Wilcoxon signed rank test. Duration of rehabilitation activities was compared using the Mann–Whitney test. The coefficient of variation of the metabolic response to exercise was calculated using percentage change between baseline and mean exercise VO_2_ values for each measurement. The coefficient of variation of baseline VO_2_ within participants was calculated using raw VO_2_ values for all exercise sessions for that participant. A two-tailed level of p<0.05 was considered statistically significant. Statistical analyses were performed by SPSS V.28 for Windows (IBM, Inc., Chicago, USA).

### Patient and public involvement (PPI)

A specific respiratory PPI group, including members who had experienced physical rehabilitation when mechanically ventilated, was involved in the design of this study; members recognised the importance of the research and agreed with the proposed methods. The group codesigned patient-facing materials, including patient information sheets.

## Results

### Recruitment

**Figure 2:**
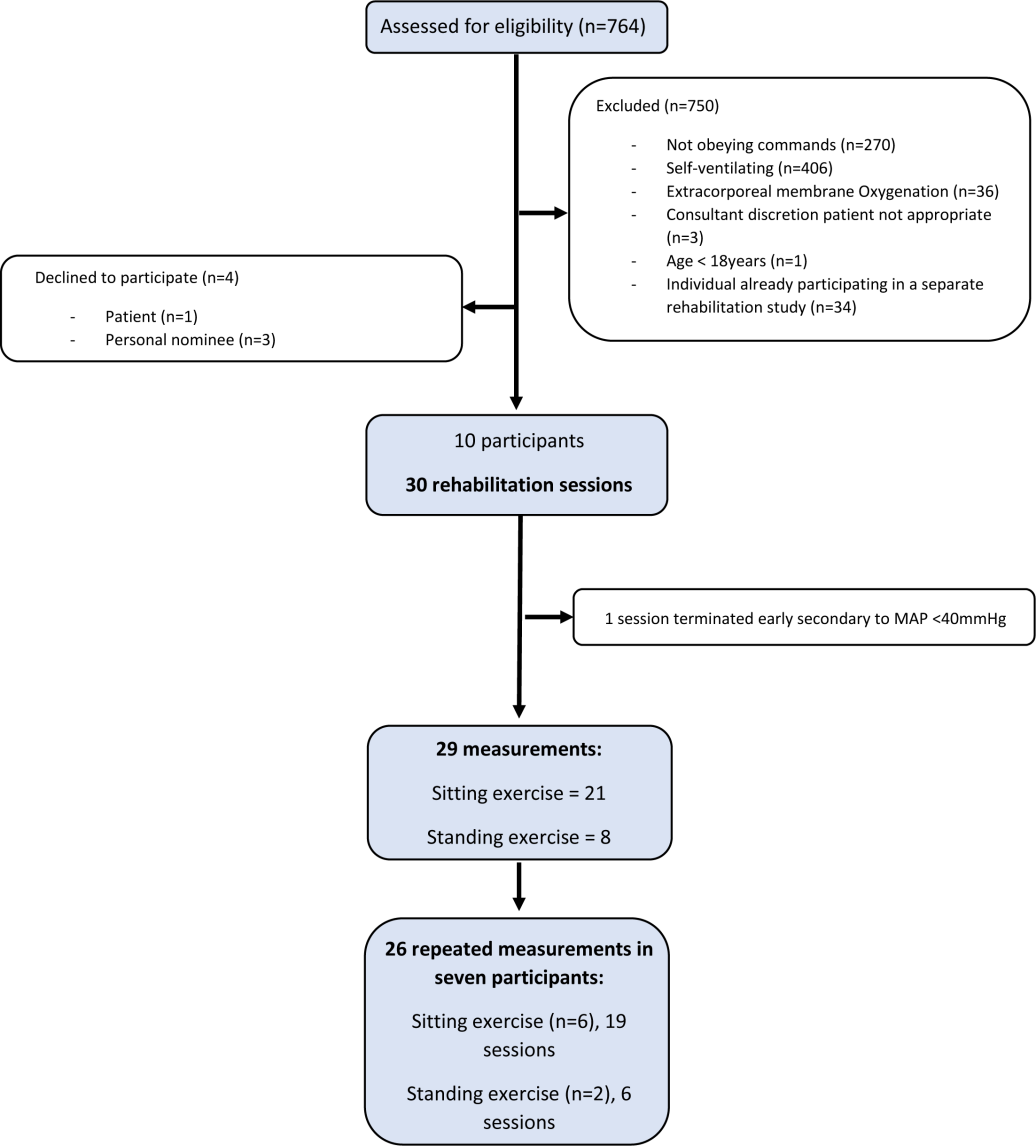
Flow of participants. MAP, mean arterial pressure.

The flow of participants is presented in figure 2. The ten participants were recruited to the study, with a total of 29 successful measurements taken during physical rehabilitation. Exercise and percentage change in VO_2_ could not be calculated in one additional measurement as the session was terminated early due to mean arterial pressure <40 mmHg (5.3 kPa) soon after the participant started rehabilitation. Main characteristics and physiological values of patients are presented in table 2; detailed patient diagnoses are provided in table 3.

**Table 2:** Baseline demographic and physiological characteristics

Variables	n=10
Sex (male: female)	7:3
Age (years)	65.5 (57.3–74.3)
Estimated body weight (kg)	76.0 (63.4–91.3)
BMI (kg/m^2^)	26.6 (21.6–28.7)
Length of MV prior to first study measurement (days)	16.5 (13.7–25.8)
APACHE II score (admission)	27.0 (25.7–32.5)
SOFA score	8.0 (6.5–8.0)
CRP (mg/L) (n=9)	83.0 (26.5–132.0)
P/F ratio (mm Hg) (kPa) (n=9)	332.5 (274.3–377.5) (44.3 (36.6–50.3))
Vasopressor inotrope score	0 (0–4)
Barthel index	20 (20–20)

Data presented as median (IQR).

APACHE IIacute physiology and chronic health evaluation scoreBMIbody mass indexCRPC-reactive proteinP/F ratioratio of partial pressure of oxygen in arterial blood to the fraction of inspiratory oxygen SOFAsequential organ failure assessment

**Table 3:** Detailed patient diagnoses

	n (%)
Medical	
Heart failure	2 (20)
Surgery	
Aortic surgery +/−valve replacement/repair	2 (20)
Heart valve replacement	4 (40)
Coronary artery bypass graft +/−valve replacement	2 (20)

### Rehabilitation sessions

#### Activity characteristics

There were 21 measurements made while participants performed sitting exercise, and 8 while performing standing exercise. The median (IQR) duration of sitting activities was 12.9 (9.6–16.1) min and 15.7 (10.5–22.1) min for standing exercises (p=0.187). Reasons for ceasing rehabilitation sessions were clinician opinion (n=15) and at the patient’s request due to fatigue (n=14).

### Metabolic response to exercise

First session VO_2_ response to exercise is detailed in table 4.

**Table 4:** First session VO_2_ (absolute and percentage) change

Exercise type	Baseline VO_2_ (mL/kg/min)	Exercise VO_2_ (mL/kg/min)	VO_2_ percentage change	P value
All (n=10)	3.54 (2.95–3.91)	4.37 (3.96–5.14)	28.5 (18.3–65)%	0.005
Sitting (n=8)	3.54 (2.91–4.20)	4.42 (3.96–5.69)	28.5 (21.5–85.7)%	0.012
Standing (n=2)	2.97 and 3.77	4.01 and 4.31	14.1% and 34.7%	–

Data presented as Mmedian (IQR) except in standing (n=2).

Median (IQR) peak VO_2_ was 4.81 (4.43–6.86) mL/kg/min and was significantly different to baseline (pp<0.001).

**Figure 3:**
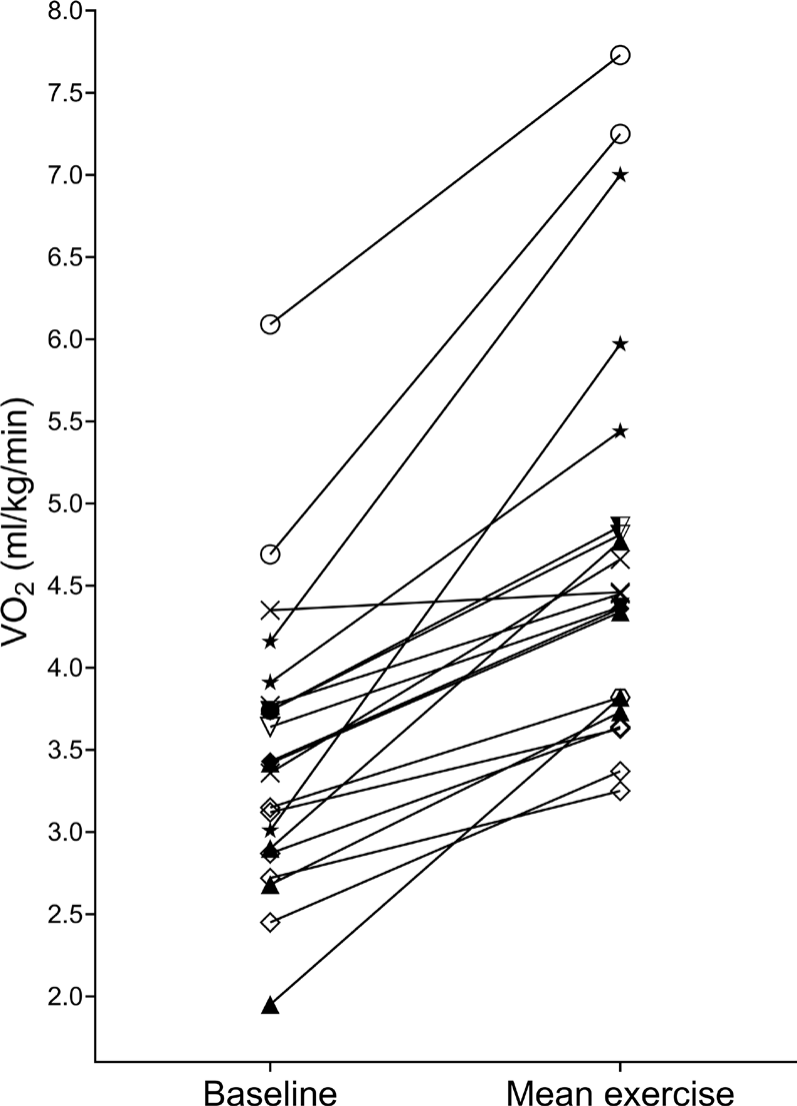
Response to exercise during sitting activities (baseline and mean exercise VO_2_) in 21 sessions. Shapes depict individual participants; seven participants completed >1 sitting exercise session.

The response to sitting and standing exercise during all rehabilitation sessions is detailed in figures3  4, respectively.

**Figure 4:**
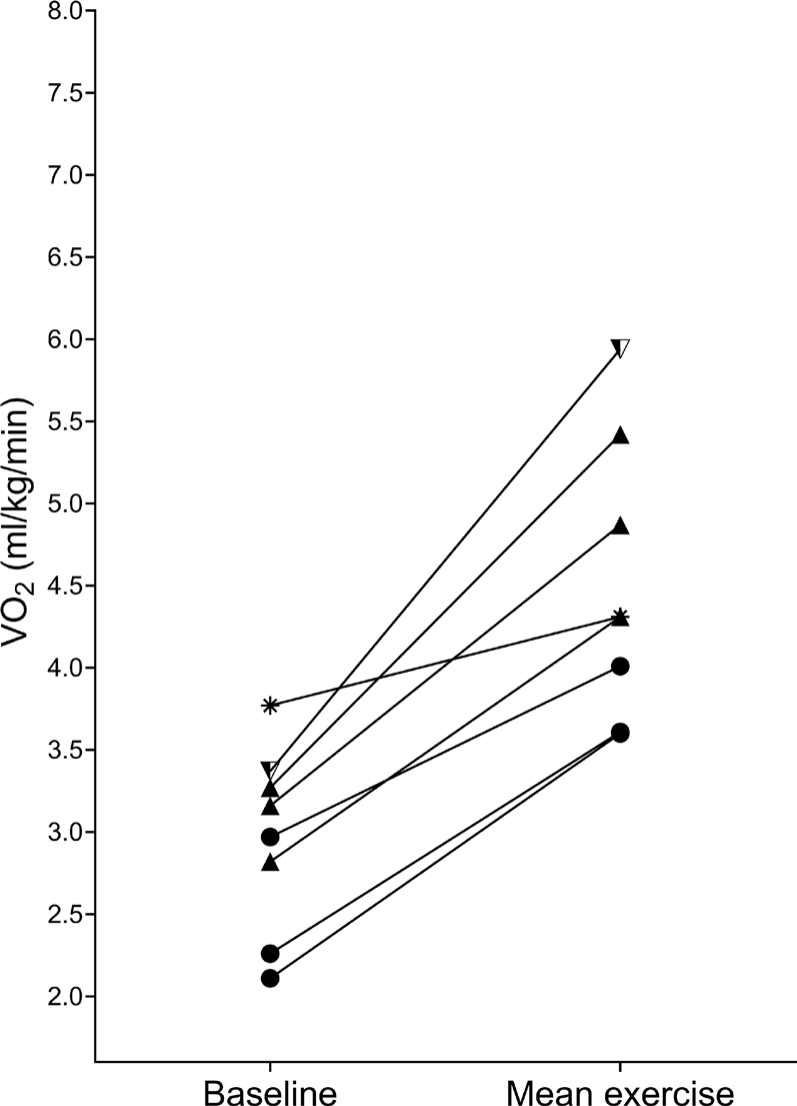
Response to exercise during standing activities (baseline and mean exercise VO_2_). Shapes depict individual participants; two participants completed >1 standing exercise.

### Variability of the metabolic response to exercise within participants

The percentage change in VO_2_ from baseline to mean exercise varied considerably within participants. The median (IQR) within-patient coefficient of variation of percentage change in VO_2_ in participants (n=7) who completed more than one rehabilitation session (range 2–7 sessions) was 43 (34%–61)% in 26 measurements.

**Figure 5:**
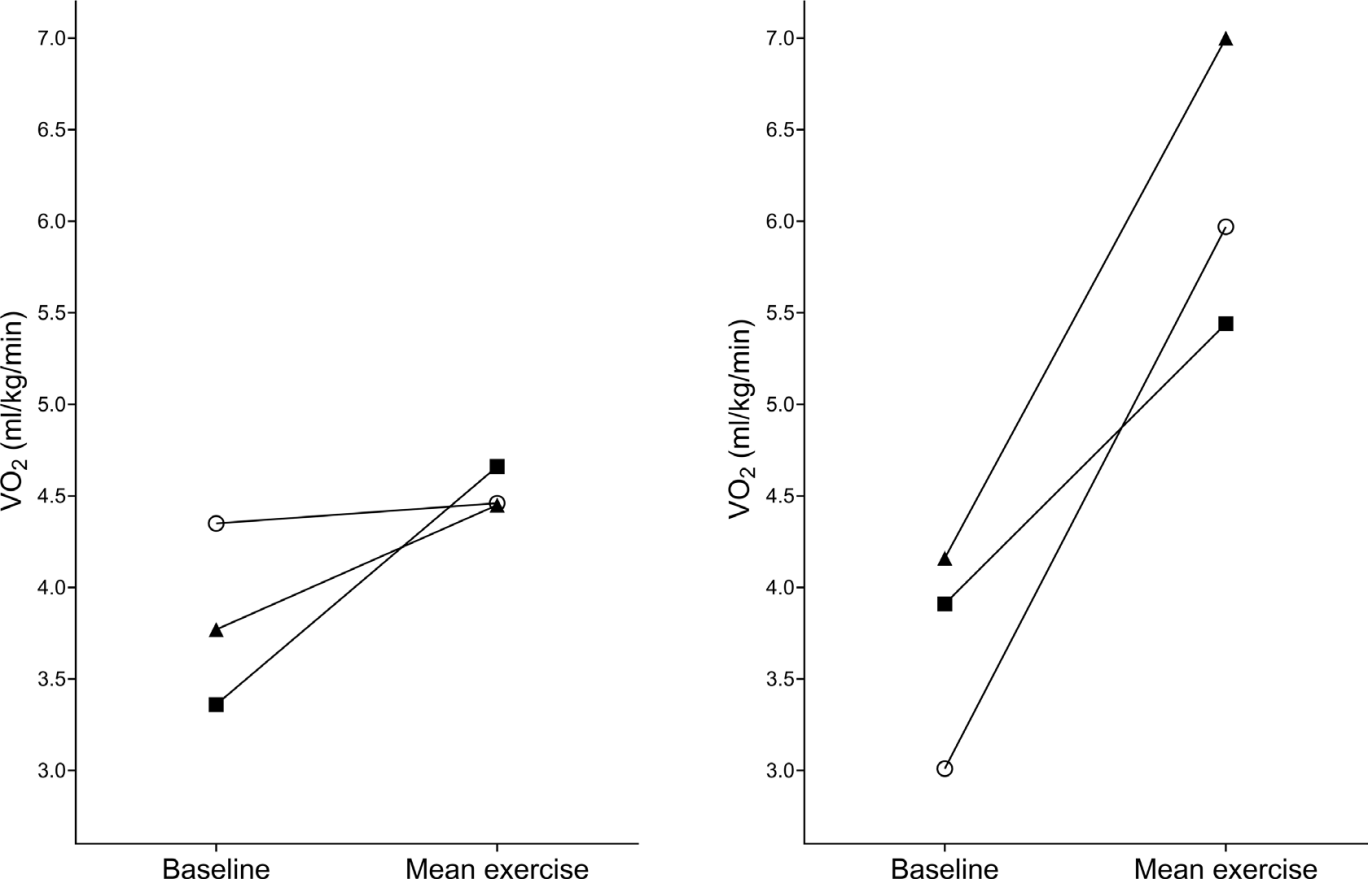
Response to sitting exercise in participant 3 (left) and participant 7 (right). Circle=session 1, triangle=session 2 and square=session 3.

Median (IQR) within-patient coefficient of variation of percentage change in VO_2_ was 46 (26%–63)% in participants performing >1 sitting exercise session (6 participants, 19 sessions). Two participants performed >1 standing exercise session (six sessions) showing a within-patient coefficient of variation of 12% and 33%. The exercise responses of participants 3 and 7 during sitting exercise are shown graphically in figure 5.

### Variability of baseline VO_2_ within participants

Baseline VO_2_ (mL/kg/min) also varied within participants. The median (IQR) within-patient coefficient of variation of baseline VO_2_ in participants (n=7) who completed more than one rehabilitation session (range 2–7 sessions) was 16 (10 to 18)%. The median (IQR) within-patient coefficient of variation of baseline VO_2_ was 15 (8 to 19)% in participants performing >1 sitting exercise session (6 participants, 19 sessions). Two participants performed >1 standing exercise session (six sessions) showing a within-patient coefficient of variation of baseline VO_2_ of 8% and 19%.

## Discussion

We present data documenting the variability in the metabolic cost of rehabilitation in mechanically ventilated patients in critical care. While physical rehabilitation significantly increases VO_2_ compared with baseline, the response varied considerably within and between participants.

### Feasibility of measuring VO_2_ using the Beacon caresystem during physical rehabilitation

Performing breath-by-breath measurement of VO_2_ in critically ill patients can be challenging, with other authors reporting numerous unusable measurements.^12 19^ However, we found the measurement of VO_2_ using the Beacon Caresystem feasible with no technical issues during exercise and no loss of data at the analysis stage. Despite this, the system only provides approximate VO_2_ data in real time; further, offline analysis was required to calculate exact values.

### Variability in the metabolic response to exercise

Our data are in agreement with other studies, which also found considerable variation in VO_2_ change *between* patients undertaking the same functional activity, as demonstrated by wide 95% CIs and interquartile ranges in their data,^12 14 15 17^ and reported, but not fully quantified in one study.^19^ However, we have also quantified the variability of VO_2_ change within participants.

The complexities of rehabilitation in critical care mean that many factors will affect the VO_2_ response to exercise in critically ill patients. Physiologically, the stage of critical illness and the level of ICU-acquired weakness will be important. Mitochondrial dysfunction associated with critical illness prevents ATP production and will affect patients’ ability to generate sufficient substrate to perform effective muscle contraction.^23^ Sedation, delirium, alertness and pain will all influence a patient’s ability to engage in exercise.^23 24^ Although all patients in the current study were able to consistently obey commands, these factors will affect VO_2_ change during physical rehabilitation.^23 24^

**Figure 6:**
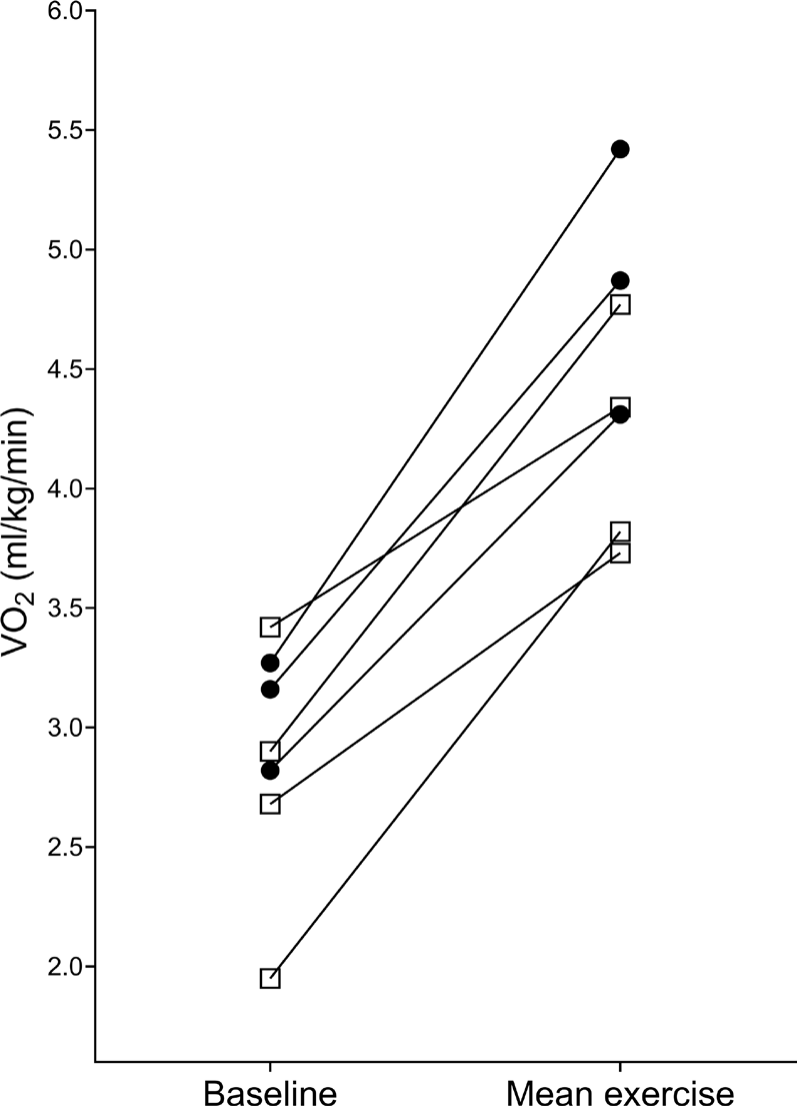
VO_2_ response to exercise in participant 8. Square depicts sitting exercise and circle depicts standing exercise.

As an example, one participant in our study (figure 5, left) increased their VO_2_ by only 2.5% during sitting exercise; further examination of physiotherapist records from this session reports minimal patient participation and significant support in sitting from the therapist. In subsequent sessions, the participant’s VO_2_ increased more despite remaining in the ICU mobility score category of 3. Furthermore, we found that some participants had a higher VO_2_ percentage increase during sitting exercise than in standing exercise; this was observed between participants (figure 4) and within one participant in our dataset (figure 6). Existing data also suggest that there is significant overlap in the VO_2_ response between sitting and standing exercise.^19^ The ICU mobility scale is frequently used in research studies and practice to objectively quantify or progress the level of mobilisation, perhaps with the assumption that a higher ICU mobility score relates to an increased intensity of exercise. However, our data indicate that the ICU mobility scale does not necessarily relate to oxygen consumption and could explain why protocolised rehabilitation interventions or interventions targeting the highest level of mobilisation as indicated by a functional measure may not improve outcomes after critical illness.^7 8 11^ Perceived intensity (such as the Borg scale) may bear a stronger relationship with VO_2_, but factors such as fatigue, drowsiness and patient understanding mean that this is a difficult measure to complete in this complex population.

The VO_2_ change found in our data mirrors the findings of previous similar work in the field. Black *et al* found a mean (SD) percentage increase in VO_2_ of 23 (11.2)% during sitting exercise and 34.8 (13.3)% in standing exercise but did not report raw VO_2_ values,^19^ making it impossible to directly compare VO_2_. Tipping *et al* reported comparable baseline VO_2_ (262.33 mL/min (95% CI 201.97 to 322.70)) and exercise VO_2_ (353.02 mL/min (95% CI 303.50 to 402.55))^13^ to our data (when unadjusted for weight) during sitting exercise in a small sample of ten exercise sessions, but dissimilar to our data, exercise VO_2_ measurement was started after the participant achieved the upright sitting position; it is likely that the participant expended considerable energy transferring from the lying to sitting position without this being captured in the VO_2_ analysis. Other studies measuring VO_2_ response during exercise in mechanically ventilated patients only did so during bed exercises and cycling in bed^1215^,^17^ or during mixed physiotherapy interventions, including passive exercise.^18^

### Implications for future research

It is important to highlight that the rehabilitation of critically ill patients is complex and requires more than purely physiological assessment. Multidisciplinary, individualised interventions, taking into consideration patient goals and preferences, previous function and other patient, family and staff priorities must be offered, but in order to maintain or improve muscle mass, strength and cardiovascular fitness, overloading of the body systems is required, which requires an increase in VO_2_.^23 25^

Despite growing data describing VO_2_ during physical rehabilitation in critical care, the optimal exercise intensity in mechanically ventilated patients remains unclear.^17^ Data show that mechanically ventilated individuals subjected to a formal incremental cycling exercise test reached only 23.1–55.2% (median 34.3%) of their predicted VO_2_max at peak exercise, but reached 76%–89% of their achieved VO_2_peak within the first minute of unloaded cycling.^17^ For any athlete, exercising at 80% of VO_2_peak would be considered high-intensity training,^26^ suggesting that these patients were exercising at high intensity for a majority of their exercise test (which averaged 8 min).^17^ These data, and significant variability in energy requirements between and within critically ill individuals undertaking the same functional activity present in our and others’ data, suggest that the simplistic measures of function and/or exercise intensity (such as the ICU mobility scale) are inadequate in the critically ill patient. Given the inconsistent benefits from the existing interventional rehabilitation trials in critical care, along with exercise limitations in this patient group,^17^ formal cardiopulmonary exercise testing could have the potential to truly guide exercise interventions in mechanically ventilated patients and categorise exercise interventions in future interventional studies.^27 28^ While challenging, cardiopulmonary exercise testing is feasible in the critically ill patient, but further advances in technology and software are needed to enable the accurate ‘real-time’ measurement of oxygen consumption during the rehabilitation session itself.^17^

#### Critique of the method

This study has some limitations. It is important to acknowledge that the population of patients studied was recruited from specialist medical, surgical and transplant ICUs with underlying heart disease as a primary reason for admission and a long duration of MV before they were eligible to participate in the study, meaning that it is not necessarily possible to extrapolate these findings to all mechanically ventilated patients in ICU. The small number of eligible screened patients (owing to neurological compromise, sedation and ECMO) highlights the challenging reality of performing physical rehabilitation in mechanically ventilated patients with persistent critical illness. Measurement of perceived exertion may have provided more insight into VO_2_ changes between sessions, but owing to fatigue, drowsiness and patient understanding, we were only able to obtain these data for three sessions, meaning that they were not included in the analysis. It must, however, be noted that all participants were encouraged to achieve their maximal level of activity in each session. The small sample size and skewed data meant that it was not possible to undertake more complex, integrated analysis incorporating all data points into a single model or to correct our analysis for other factors which could potentially influence VO_2_, such as sedation or pain scores, SOFA, CRP or P/F ratio.

## Conclusions

This study has given further insight into the physiological demands of physical rehabilitation in critically ill patients. Our data show that VO_2_ change is highly variable between and within individuals performing the same functional activity, indicating that functional measures such as the ICU mobility scale do not necessarily relate to oxygen consumption. Cardiopulmonary exercise testing and measurement of VO_2_ during exercise may have the potential to assist with exercise prescription and categorisation of rehabilitation interventions in mechanically ventilated patients in the future.

## Data Availability

Data are available on reasonable request.
